# Exploring system drivers of gender inequity in development assistance for health and opportunities for action

**DOI:** 10.12688/gatesopenres.13639.2

**Published:** 2023-07-17

**Authors:** Doris Bartel, Amanda Coile, Annette Zou, Adolfo Martinez Valle, Hester Mkwinda Nyasulu, Logan Brenzel, Nosa Orobaton, Sweta Saxena, Paulina Addy, Sita Strother, Modupe Ogundimu, Banny Banerjee, Dyness Kasungami

**Affiliations:** 1 Independent, Washington, District of Columbia, USA; 2 JSI Research and Training Institute, Inc., Arlington, Virginia, 22202, USA; 3 Global ChangeLabs, Portola Valley, California, 94028, USA; 4 Health Policy and Population Research Center (CIPPS), Universidad Nacional Autónoma de México, Mexico City, 04510, Mexico; 5 White Ribbon Alliance, Malawi, Lilongwe, Malawi; 6 Bill & Melinda Gates Foundation, Seattle, Washington, 98109, USA; 7 U.S. Agency for International Development (USAID), Washington, District of Columbia, 20523, USA; 8 Women in Agricultural Development, Ministry of Food and Agriculture, Accra, Ghana; 9 National Health Insurance Scheme, Abuja, Nigeria

**Keywords:** gender, gender inequity, development assistance for health, system analysis, co-creation, power, gender transformative, health system

## Abstract

**Background** ***:*** Deep-rooted and widespread gender-based bias and discrimination threaten achievement of the Sustainable Development Goals. Despite evidence that addressing gender inequities contributes to better health and development outcomes, the resources for, and effectiveness of, such efforts in development assistance for health (DAH) have been insufficient. This paper explores systemic challenges in DAH that perpetuate or contribute to gender inequities, with a particular focus on the role of external donors and funders.

**Methods:** We applied a co-creation system design process to map and analyze interactions between donors and recipient countries, and articulate drivers of gender inequities within the landscape of DAH. We conducted qualitative primary data collection and analysis in 2021 via virtual facilitated discussions and visual mapping exercises among a diverse set of 41 stakeholders, including representatives from donor institutions, country governments, academia, and civil society.

**Results:** Six systemic challenges emerged as perpetuating or contributing to gender inequities in DAH: 1) insufficient input and leadership from groups affected by gender bias and discrimination; 2) decision-maker blind spots inhibit capacity to address gender inequities; 3) imbalanced power dynamics contribute to insufficient resources and attention to gender priorities; 4) donor funding structures limit efforts to effectively address gender inequities; 5) fragmented programming impedes coordinated attention to the root causes of gender inequities; and 6) data bias contributes to insufficient understanding of and attention to gender inequities.

**Conclusions** ***:*** Many of the drivers impeding progress on gender equity in DAH are embedded in power dynamics that distance and disempower people affected by gender inequities. Overcoming these dynamics will require more than technical solutions. Groups affected by gender inequities must be centered in leadership and decision-making at micro and macro levels, with practices and structures that enable co-creation and mutual accountability in the design, implementation, and evaluation of health programs.

## Introduction

Deep-rooted and widespread gender-based bias and discrimination threaten the achievement of the Sustainable Development Goals (SDGs) ( https://sdgs.un.org/goals), including ensuring healthy lives and wellbeing of people at all ages and gender equality as a fundamental human right ^1^. Here, gender refers to the culturally defined attributes, entitlements, responsibilities, and expectations associated with being, or being perceived as, feminine/woman/girl, masculine/man/boy ^2^ or non-binary/genderqueer ^3^. Gender is driven by a social and structural stratification system of power distribution and patterned behaviors, that manifest at the individual, interactional, and macro levels ^4^.

Gender is one of many social determinants that contribute to health and development outcomes ^5, 6^. Gender norms can shape institutional systems and practices, including whether and how the health needs of certain groups of people are acknowledged, whether they can access resources such as health care, and whether they can realize their choices and rights ^7^. Gender bias and discrimination in institutions and national health systems enable practices and policies that produce inequitable health and gender outcomes ^8– 12^. These inequities are socially produced, systematic in their distribution, avoidable, unfair, and unjust ^7^.

A growing body of evidence suggests that eliminating or mitigating gender and health inequities contributes to better health and development results ^2, 13– 16^. However, despite decades of global commitments and advocacy by women’s groups and scholars, resources and effectiveness of efforts to reduce gender inequities in development assistance for health (DAH) ^a^ investments have been limited or insufficient ^17– 20^.

Scholars and feminist activist groups are asking why actions are weak, resources small or ineffective, and progress is slow ^2, 12, 16^. A complex and multifaceted set of contributing factors is possible. For example, recent studies show that global health institution accountability for and implementation of gender policies and practices are inadequate ^20– 28^. Gender bias and inequities pervade the leadership, organizational structures, and culture of global health institutions such as donors, international nongovernmental organizations (INGOs), and multilateral agencies ^29^. Furthermore, some studies suggest that broader system dynamics and power asymmetries between actors in DAH play a role in shaping the way that health systems are conceptualized, funded, governed, and implemented ^30– 33^, and can inadvertently reinforce gender and health inequities ^20^.

There is growing recognition within the global health community that complex and protracted challenges such as gender and health inequities require a deeper understanding of the linkages, relationships, interactions, and behaviors of such actors ^34, 35^. While there has been significant research in how system dynamics and power asymmetries between actors in global health aid play a role in shaping health systems, no studies, to our knowledge, have examined the drivers of gender inequity across the broader landscape of DAH. ^b^

### A systems approach to gender and health inequities

Systems theory, an interdisciplinary field of science that analyzes the dynamic interactions of interrelated, interdependent parts that make up a complex whole, has gained attention as relevant for health systems analysis and interventions ^37^. Application of systems theory can benefit the exploration of macro-level dynamics affecting complex and protracted issues, making it a useful basis for exploring the drivers of gender and health inequities in DAH ^37– 39^. Systems approaches have been used in social intervention research, such as studies examining interventions that tackle intimate partner violence ^40, 41^. However, there are relatively few studies that use a systems approach to analyze progress in minimizing gender and health inequities. Moreover, the operant dynamics and drivers in the landscape of DAH that reinforce gender bias are poorly documented.

### Framing and purpose of this paper

This paper builds on prior work, and is the third manuscript stemming from a longer process examining the shifts needed in DAH to facilitate a redistribution of power, and coordination and accountability between countries and donors ^c^ in designing health technical assistance (TA) interventions ^42^. Such shifts are needed to foster more resilient health systems and sustained health outcomes ^33^. We anticipate that efforts to redistribute power in ways that center local stakeholders in decision-making and build mutual accountability cannot be fully realized without addressing gender inequities.

The objective of this paper is to identify systemic challenges in DAH that are perpetuating or contributing to gender inequities, with a particular focus on the role of external donors and funders. In this paper, we map and analyze interactions between donors and recipient countries and articulate drivers of gender inequities within the landscape of DAH. As a basis for exploring and identifying actionable steps to improve gender and health equity outcomes, we aim to highlight systemic issues that impede or slow progress in addressing gender and health inequities in DAH.

## Methods

The work presented in this paper was conducted as part of a broader initiative led by the Inter-agency Working Group (IAWG) for Capacity Strengthening ^d^ to co-create a systems understanding of capacity strengthening in the context of global DAH ^43^. The process was guided by System Acupuncture® ^e^ — a theory and method that enables the design of innovative, actionable, and synergistic interventions that drive deep and sustained transformation of system behaviors and outcomes ^49^ — and focused on the system diagnostic component of the System Acupuncture process.

### Defining the system

For the IAWG initiative and this paper, the scope of the system is defined by the complex relationships of actors and institutions interacting within the landscape of DAH, and the norms that inform their behaviors and decisions.

The System Acupuncture method asks what outcomes we want to see from the system, and uses this vision to explore why the system isn’t producing these outcomes.

**Figure 1.**
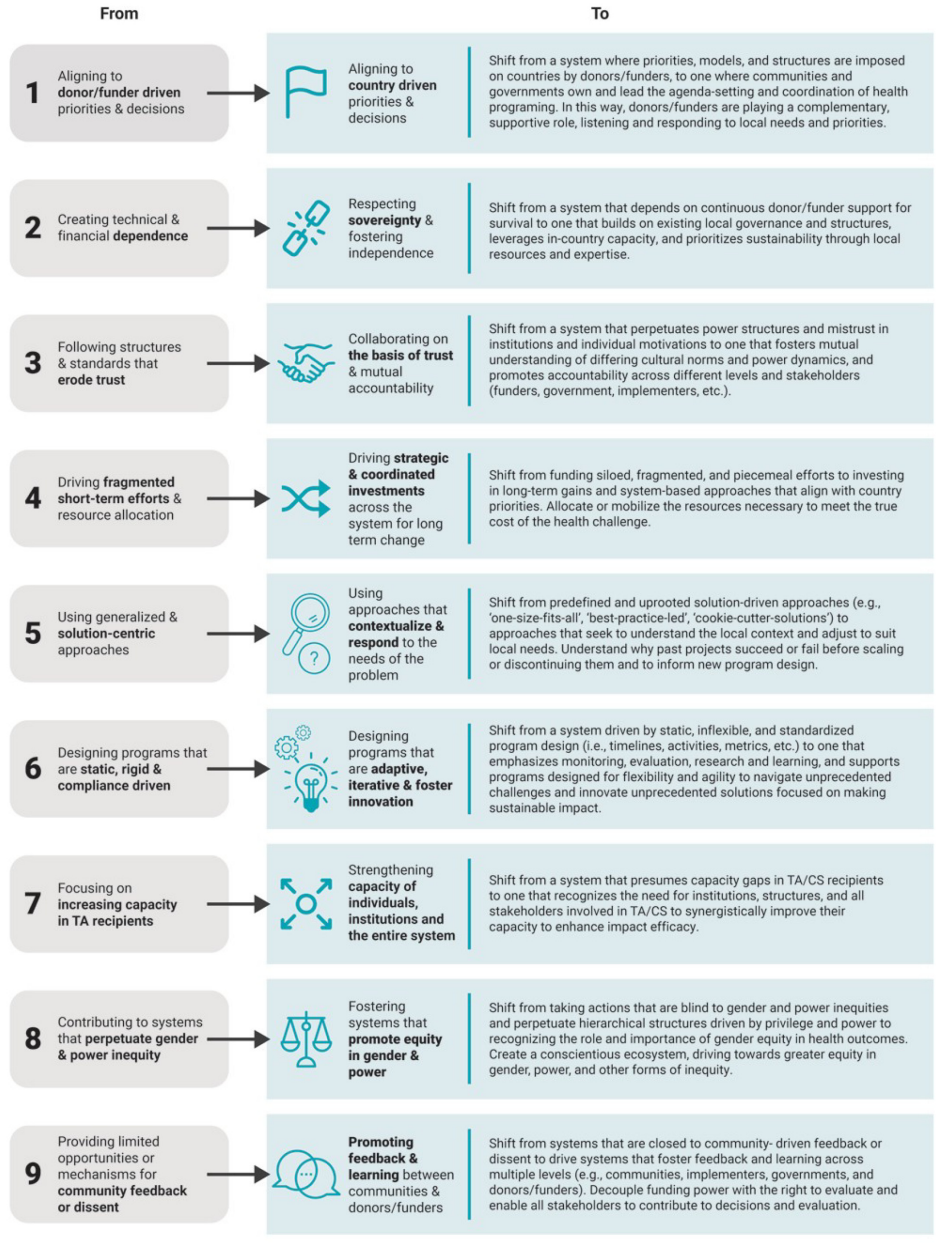
Critical shifts for capacity Strengthening.

In this case, the IAWG aligned around a set of critical shifts ( Figure 1) to represent the desired outcomes of the system. ^f^ The critical shifts, which outline a vision for more country-driven, coordinated, and equitable health investments, served as the guiding framework to co-create an understanding of the DAH system. These critical shifts originated from a previous phase of this work in DRC and Nigeria, which is explored in a separate manuscript ^42^.

The initiative’s hypothesis was that application and realization of the critical shifts by actors in the system would enhance the capacity of global health institutions to deliver sustained health outcomes. A gender lens was prioritized as part of the process in recognition that gender bias and inequities are manifest throughout the global health landscape, and the critical shifts and desired impact from health investments cannot be fully realized without addressing these factors.

### Co-creation and participant engagement

Using the methods and tools from System Acupuncture®, we took a structured process consistent with social constructivist approaches ^g^ to enable system actors to collectively understand and improve a complex adaptive system. This was facilitated through an iterative co-creation process to build a human-centered understanding of the system. In this case, we use the term co-creation to mean *an approach to creating outputs together with multiple stakeholders by leveraging their different experiences and expertise*. This was facilitated through workshops, collaborative working sessions, desk review, and key informant interviews.

**Figure 2.**
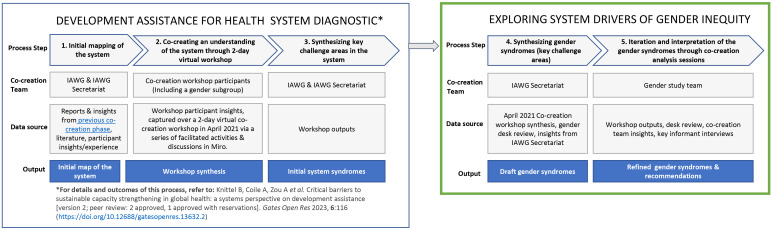
Summary of the process steps.

Given the iterative nature of this process, different actors and co-creation participants were involved at varying stages. A summary of the co-creation actors is included below; Figure 2 provides an overview of how these different actors, including the co-authors of this manuscript, were involved throughout the process.

- IAWG: The cross-agency funder working group spearheading this initiative. This group includes two representatives from USAID, three from the World Bank, and two from the Gates Foundation.
- IAWG Secretariat: The group facilitating the co-creation process and analysis. This group includes two systems specialists, two global health specialists, one gender specialist, one M&E specialist, and one coordinator.
- Co-creation workshop participants (2-day workshop in April 2021). 41 participants from 13 countries (including select IAWG representatives) Ethiopia (2 participants), Ghana (4), India (2), Kenya (5), Malawi (3), Mexico (1), Mozambique (1), Nepal (2), Nigeria (9), Uganda (2), United States (6), Zimbabwe (2), Zambia (2). The secretariat and IAWG members used purposive sampling to identify actors within their networks who could bring diverse perspectives on how health funding and TA is structured at various levels and how donor processes, models, and norms constrain or amplify health system capacity strengthening and sustainable health outcomes. Participants were selected based on their availability and to ensure diversity in background, institutional affiliation, geography and perspectives in order to co-create a systems view. Civil society organizations representing women’s issues were explicitly included.
- Co-creation workshop gender sub-group. Subset of six participants facilitated by a gender expert, who specifically explored dynamics and drivers related to gender and health inequities that are slowing or impeding progress in DAH. This sub-group included: a government representative from Ghana, two government representatives from Nigeria, a civil society implementer from Malawi, a public health academia representative from Mexico, and a U.S.-based donor representative (from the IAWG).
- Gender study team. The secretariat solicited interest from the six gender sub-group co-creation participants and the IAWG members to form a gender study team to further interrogate the system drivers of gender inequity that emerged from the co-creation workshop. All those who participated in the analysis and writing of the paper were included as co-authors. This team included five of the six original co-creation workshop gender sub-group participants, two additional IAWG representatives, and five secretariat members.

### Data collection and analysis

**Primary data:** Primary data were collected and analyzed via facilitated discussions and visual mapping exercises, through an iterative virtual engagement, facilitated by the secretariat, over nine months in 2021. System maps were used to co-create, describe, and visualize the multi-dimensional view of causal connections between individual drivers in the system. ^h^ Drivers refer to identifiable forces (i.e., structural, policy, and funding decisions or behaviors) that can influence different elements of the system to act in specific ways (in this case, perpetuating gender inequities in the DAH landscape).

The process aimed to facilitate a shared understanding of system dynamics across the DAH landscape and articulate and clarify the perspectives and experiences of a diverse set of actors, including donors, national government ministry representatives, academia, and civil society. 

**Desk Review:** We also conducted discussions with the IAWG and completed an iterative and non-systematic literature review to inform the system mapping process. The literature review was based on Google Scholar and PubMed searches using multiple permutations of search terms: gender, power, social determinants, social accountability, development assistance for health, donor, and health system. An iterative approach was applied, refining terms and adding articles from sources cited as the review proceeded. Sources were selected on the basis of relevance to the topic of gender, power, and development assistance for health. The literature review was limited to English language sources from the years 2000 to 2022. In total, 52 peer-reviewed journal articles and nine relevant reports and commentaries were reviewed. Systematic analysis of the sources included thematic coding for themes based on questions driving the review, including: (1) How do gender bias, discrimination and power dynamics manifest in national health systems, and how do gender inequities contribute to poor health?; (2) How do gender bias, discrimination and power dynamics manifest in the landscape of donor assistance for health?; (3) How does donor assistance for health programming succeed or fail to support attention to gender bias and discrimination?

**Process steps:** Figure 2 provides a visual summary of the overall process. The process steps included:

1. Initial mapping of the system: An initial system map was first created by the IAWG and IAWG secretariat, drawing on professional insights and experience, documentation from a previous phase of work, and additional literature, including a preliminary set of drivers related to gender and health inequities, as a starting point. 

2. Co-creating an understanding of the system: The map was then expanded upon through a virtual co-creation workshop held over two days in April 2021.

During the workshop, participants used a collaborative virtual whiteboard tool to capture and depict specific behaviors, dynamics, and characteristics of donor and country stakeholder group interactions in the DAH system. A sub-group of participants (‘co-creation workshop gender sub-group’) specifically focused on system dynamics that hinder progress to reduce gender and health inequities, as they experienced them in their context. The workshop was facilitated through a series of virtual worksheets where participants added insights individually and then discussed and refined them as a group.

3. Synthesizing key challenge areas in the system: The Secretariat utilized the workshop outputs to identify patterns of dynamics in the system. Participant insights were synthesized by grouping key drivers into thematic challenge areas and spatially arranging the drivers into three broad contexts based on where the policy or funding decisions, behaviors, or actions occur: the donor space, the country space, and the space where they intersect. This initial synthesis resulted in the identification of systemic challenges inhibiting progress on the critical shifts. This is explored in detail in a separate manuscript ^43^. 

4. Synthesizing gender syndromes: The Secretariat then revisited the workshop content from a gender lens to explicitly highlight systemic challenges in DAH that perpetuate or contribute to gender inequities. While this drew most heavily from the workshop outputs generated by the gender sub-group, the synthesis also drew related content from across all workshop sub-groups. Insights from the desk review informed the interpretation, prioritization, and grouping of participant inputs. The synthesis resulted in the identification and mapping of six gender-focused syndromes.

5. Iteration and interpretation of the gender syndromes: The co-authors of this paper, including five of the six original co-creation workshop gender sub-group participants, two additional IAWG representatives, and five secretariat members came together through two follow-on virtual co-creation analysis sessions to review, iterate, and expand on these six syndromes. This included making sense of the insights, articulating key messages, and developing conclusions and recommendations based on the findings. In addition, three semi-structured key informant interviews were held with gender experts with expertise in DAH, gender institutional capacity-strengthening or health system capacity strengthening, to further validate the findings. The key informants, recruited by the authors through a purposive sample, included two based in the global South and one in the global North.

### Ethical statement

All data collection was carried out through a facilitated co-creation process, including one virtual workshop and a series of discussions.

In the first phase, for the co-creation workshops, oral consent to collect inputs and record sessions was obtained from all participants at the start of the workshop sessions, per standard practice for minimal risk interactions. The information obtained through the workshop elicited impersonal and anonymous input, focused on participants’ expert opinions and experience. Participants were assured of confidentiality and that all findings would be anonymized and provisions made for the protection of privacy and confidentiality of the participants and the information they provided. Participant insights were collected and analyzed with complete anonymity via a virtual whiteboard tool, and were therefore unable to be linked to a single individual. No individual interviews were conducted in this phase. We did not seek ethical approval for the first phase because we determined the activities were exempt, given that they did not constitute human subjects research as described under US HHS regulation 45 CFR 46(e)(1).

In the second phase, the JSI institutional review board deemed the process and tools exempt from full review under CFR 46.101(b)(2), which covers survey activities without identifiers or sensitive questions that could result in harm; no participants in the study were minors (less than 18 years of age). Written informed consent was obtained from participants during this phase of the initiative, since it involved meetings with a smaller group of participants and thus inputs could not be fully anonymized. Written informed consent was obtained from the key informant gender experts interviewed separately.

### Positionality statement

The majority of authors are based in the Western Hemisphere and are employed by a donor, a donor-funded organization, or a private academic institution, all of which hold varying degrees of power within DAH. These positions of privilege, in addition to our personal biases and positionalities (social, economic, cultural), influence our interpretations of the data. Each of us, and each of our institutions, exist within the system we are analyzing.

## Results

### Six syndromes that slow or impede progress in gender equity in DAH

The mapping and co-creation processes resulted in: 1) a series of conceptual maps of the drivers of gender inequities within the landscape of DAH, 2) diagrams describing potential change points, and 3) a graphic representing the participants' views on priority action steps by donors ^52^. The six syndromes that emerged from the co-creation process reveal distinct yet interconnected system dynamics driving barriers to achieving gender equity and health outcomes. The term syndrome represents a set of concurrent events that form an identifiable pattern or a group of signs and symptoms that characterize a particular abnormality. By naming the thematic areas syndromes, we ask the reader to consider a metaphor for the system as a body in need of healing. The six syndromes highlight patterns of dysfunction in the system that are badly in need of repair.

Each syndrome is depicted visually (via a system map) and through a narrative summary. The narrative and maps should be read side-by-side to enhance understanding of the system dynamics. In the graphics, each circle represents a driver in the system. The individual drivers should be understood as contributing to the broader syndrome as opposed to portraying a direct causal relationship. The circles are arranged spatially to show where the drivers are in one of three spaces: 1) donor (left); 2) country (right); and 3) interaction (middle) (i.e., between donors and funding recipients). The spatial arrangement of these drivers and their connections is designed to help the reader understand and explore where the drivers originate, and how they interact, for the ultimate purpose of identifying solutions. The thicker arrows highlight important connections in the system, including feedback loops (i.e., cyclical clusters of drivers that reinforce each other, amplifying their effect and perpetuating a set of system behaviors).

While the six syndromes are depicted separately below, they are interconnected. Thus, while many syndromes touch on overlapping themes, they are explored from different angles. Overall, the syndromes should not be interpreted to reflect the behaviors of particular donors or countries, nor as manifesting in all contexts or donor initiatives. Rather, they represent the synthesis of experiences and perceptions that surfaced through the methods described above.

**Figure 3.**
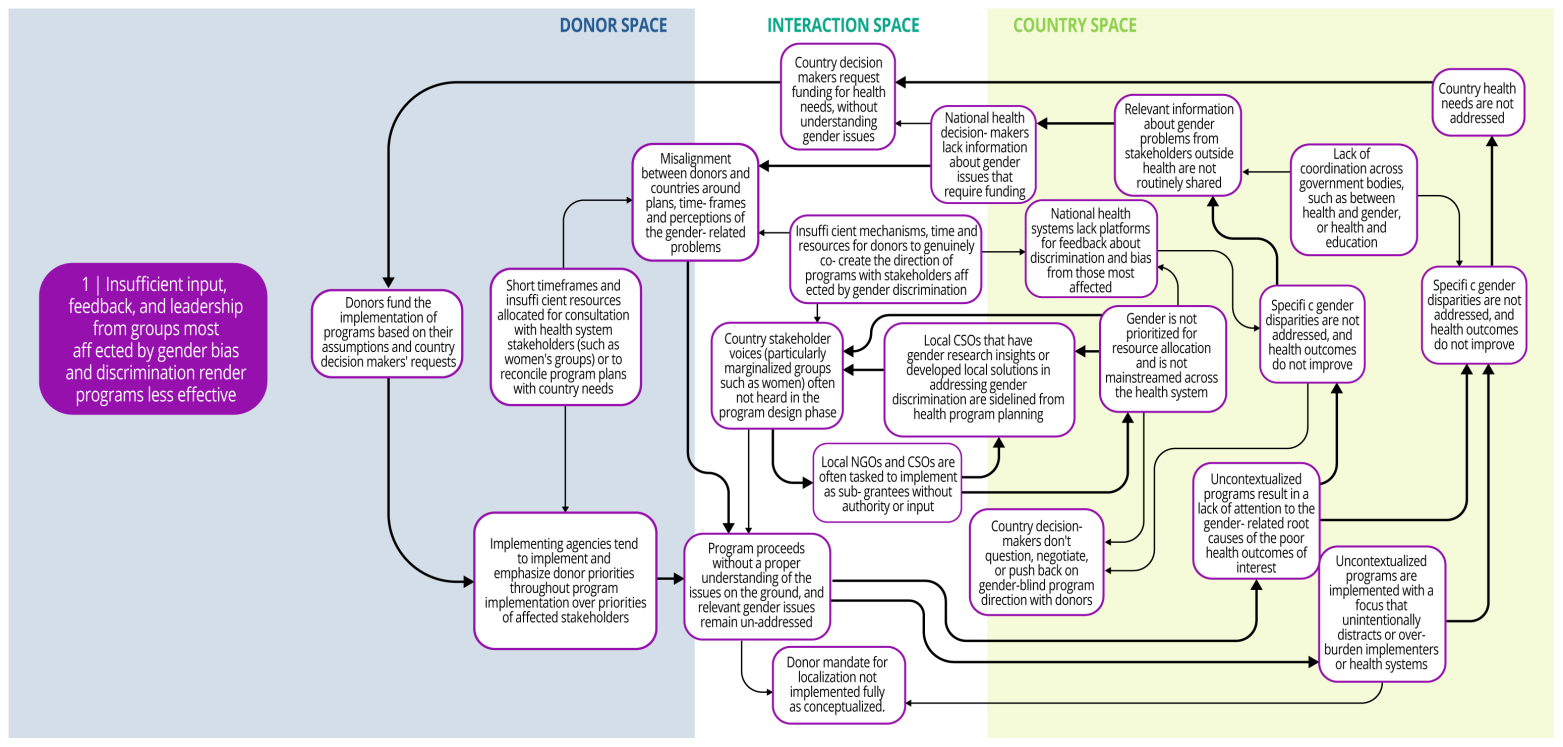
Syndrome 1: Insufficient input, feedback, and leadership from groups most affected by gender bias and discrimination render programs less effective.

*Syndrome one: Insufficient input, feedback, and leadership from groups most affected by gender bias and discrimination render programs less effective (see Figure 3)*

There are limited opportunities for community-level groups or civil society organizations with gender expertise to co-create, lead, or give feedback about DAH programming. Health programs and decisions tend to be made by national-level policy makers and technocrats, or international implementers, who often lack sufficient information about gender and health inequities. Short timeframes and insufficient resources limit opportunities for co-creation or consultation with civil society or health system stakeholders with gender expertise. Furthermore, donor funding processes and national health programs lack robust citizen engagement and mechanisms to incorporate the perspectives and leadership of local groups. In particular, women and other socially marginalized groups lack awareness of and access to platforms to voice their concerns, share pertinent information, and assume leadership roles for health system decision-making. Local civil society groups that have compiled research findings, developed local solutions, or even demonstrated achievements in reducing gender inequities in their communities may be partially or fully excluded from health program planning. Without their input and participation, health programs are designed and implemented without a full understanding of local gender and health inequities and their drivers.

**Figure 4.**
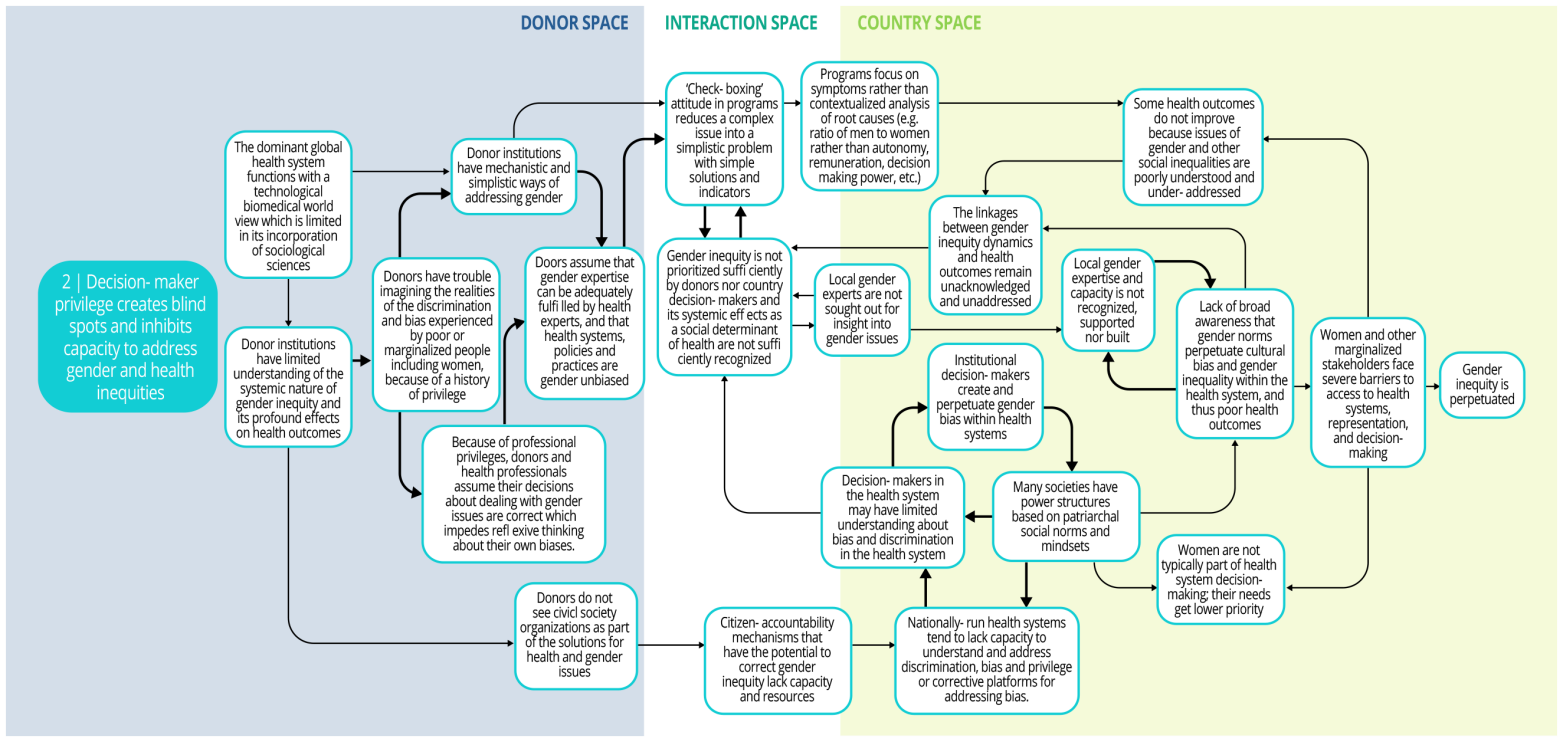
Syndrome 2: Decision-maker privilege creates blind spots and inhibits capacity to address gender and health inequities.

*Syndrome two: Decision-maker privilege creates blind spots and inhibits capacity to address gender and health inequities (see Figure 4)*

Decision-makers at high levels (whether donors, national policymakers, or technocrats) may not sufficiently prioritize actions to remedy gender disparities. One contributing factor is the influence of biases. DAH decision-makers who plan, fund, implement, and evaluate health programs often come from economic or social privilege, and their unearned privilege and power can contribute to inherent bias and blinders about gender and health inequities. For instance, decision-makers may assume that they have the expertise needed to address gender. Furthermore, a biomedical worldview, which tends to under-emphasize sociological sciences, permeates DAH. Such preconceptions can lead to overly mechanistic or simplistic ways of understanding and addressing gender in programs that fail to dismantle the root causes of inequities. The assumption that high-level health experts can remedy local gender inequities also contributes to the underuse of community gender experts, whose input is needed.

**Figure 5.**
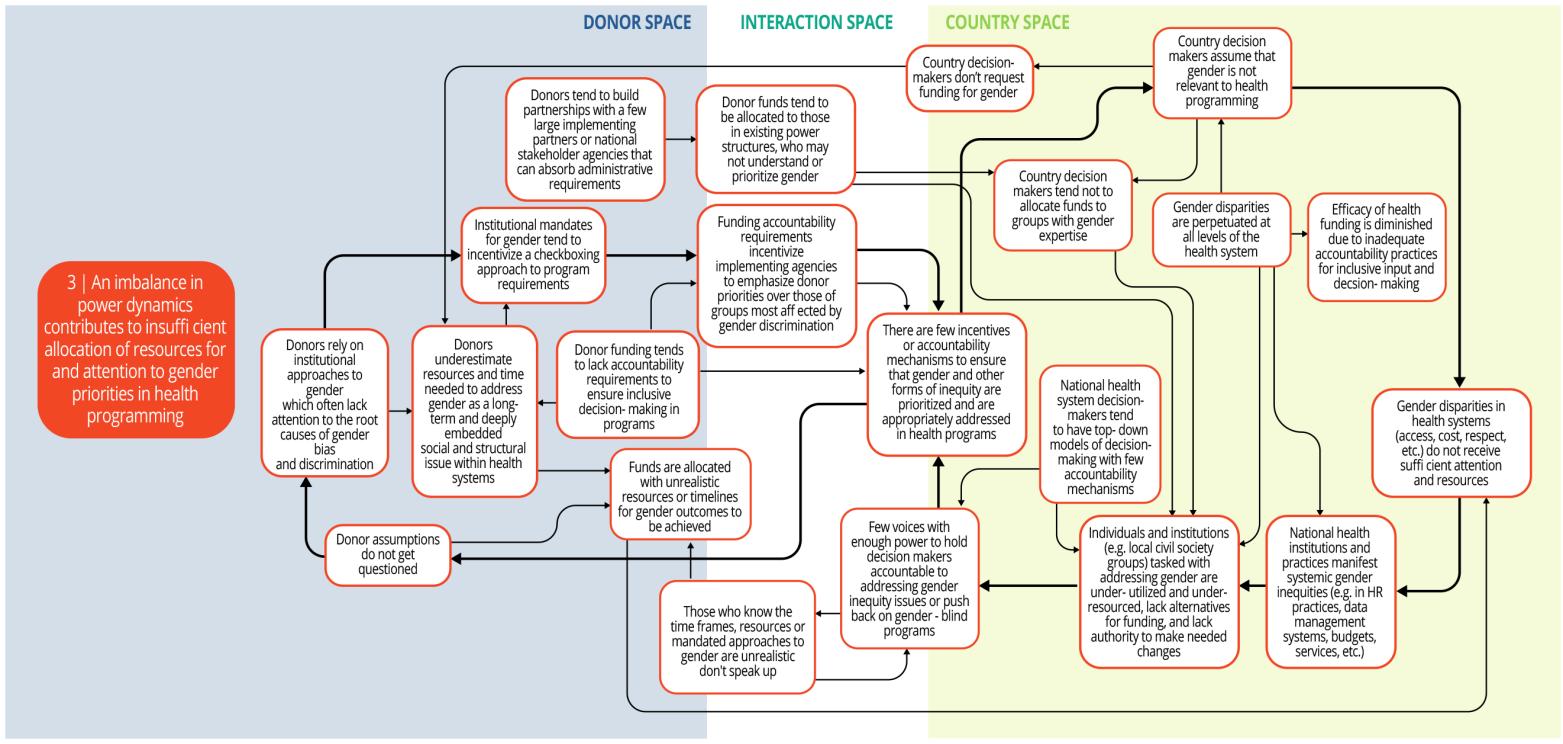
Syndrome 3: An imbalance in power dynamics contributes to insufficient allocation of resources for and attention to gender priorities in health programming.

*Syndrome three: An imbalance in power dynamics contributes to insufficient allocation of resources for and attention to gender priorities in health programming (see Figure 5)*

The imbalance of power in the funder-recipient relationship contributes to the de-emphasis of gender priorities in allocation of resources. Health institutions across the DAH landscape tend to use top-down leadership and operational models. Funding tends to be allocated to government health entities or INGOs, with accountability requirements that incentivize implementing agencies to emphasize donor priorities over those of groups most affected by gender discrimination. Donors tend to underestimate the resources and time needed to address root causes of gender and health inequities, typically relying on a ‘check-box’ approach for integrating gender in program design and measuring progress. Recipients, afraid that resources will be withdrawn, rarely question donor assumptions about timelines and costs for gender priorities. Tensions about who is making decisions, why, and for whom, exist within and among recipient organizations and are particularly acute for those that have small budgets and struggle to survive.

**Figure 6.**
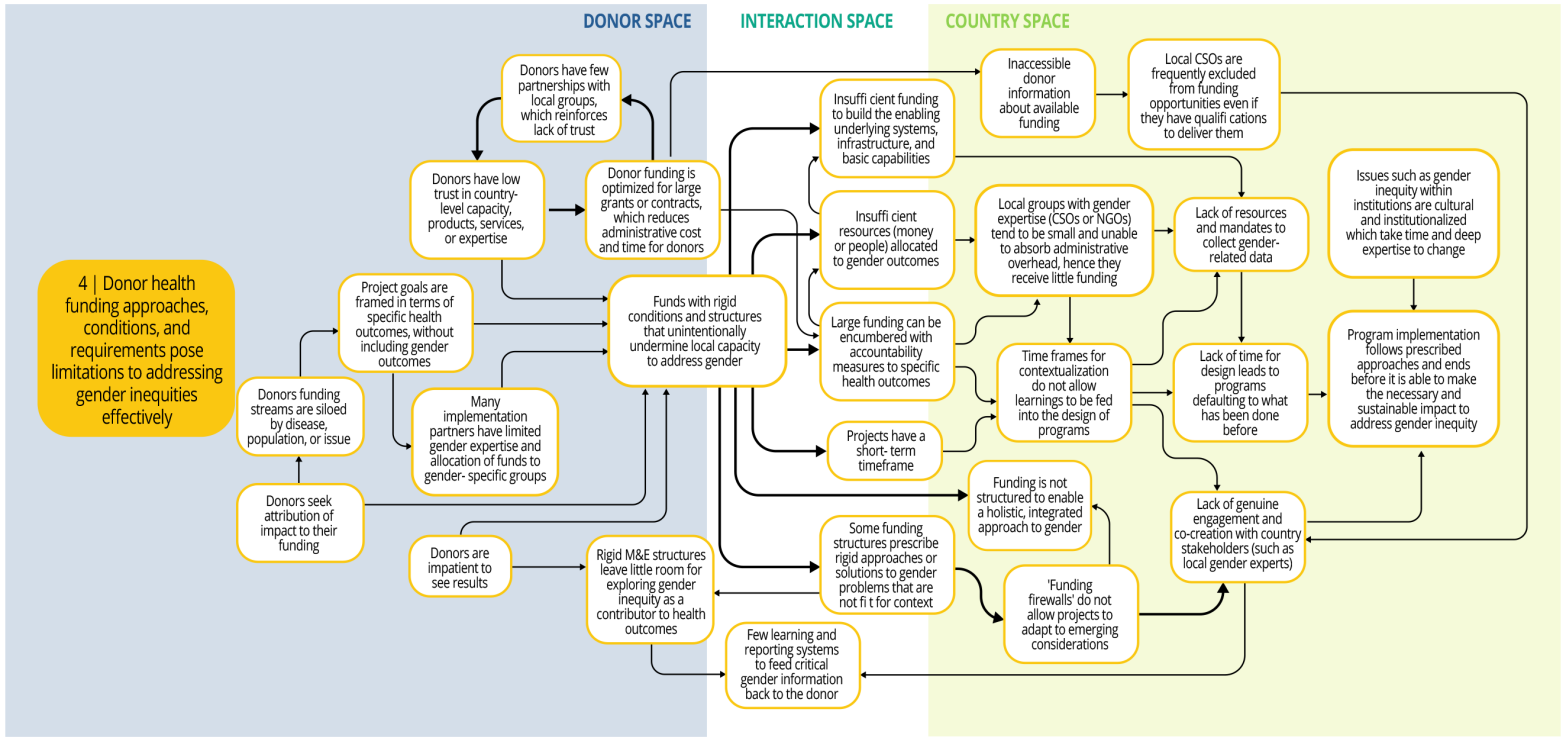
Syndrome 4: Donor health funding approaches, conditions, and requirements pose limitations to addressing gender inequities effectively.

*Syndrome four: Donor health funding approaches, conditions, and requirements pose limitations to addressing gender inequities effectively (see Figure 6)*

Funding structures for DAH can limit the efficacy of approaches to overcoming gender inequities in a number of ways. First, donor funding is structured to advantage large grants or contracts to reduce administrative costs and time and is tied to accountability measures for specific health outcomes. Local groups with gender expertise typically do not have access to information about availability of the funds or are unable to compete for this funding because of stringent accountability requirements. This contributes to a lack of genuine engagement and co-creation with local civil society organizations and stakeholders who have the requisite expertise. Stringent donor monitoring and evaluation mandates that focus on attribution of the funding to specific health outcomes leaves insufficient time and resources to track gender factors that contribute to social determinants of health. Health program reporting is typically structured for and provided directly to the donor. Critical gender inequity program information is seldom reported to decision-makers or used to share learning about gender issues with program participants and affected populations. This along with funding firewalls also limit the ability to adapt to emerging contextual changes. Beyond these factors, there is general insufficient allocation of time and resources to focus on gender outcomes in health programming and enable holistic, integrated approaches to gender in health system strengthening.

**Figure 7.**
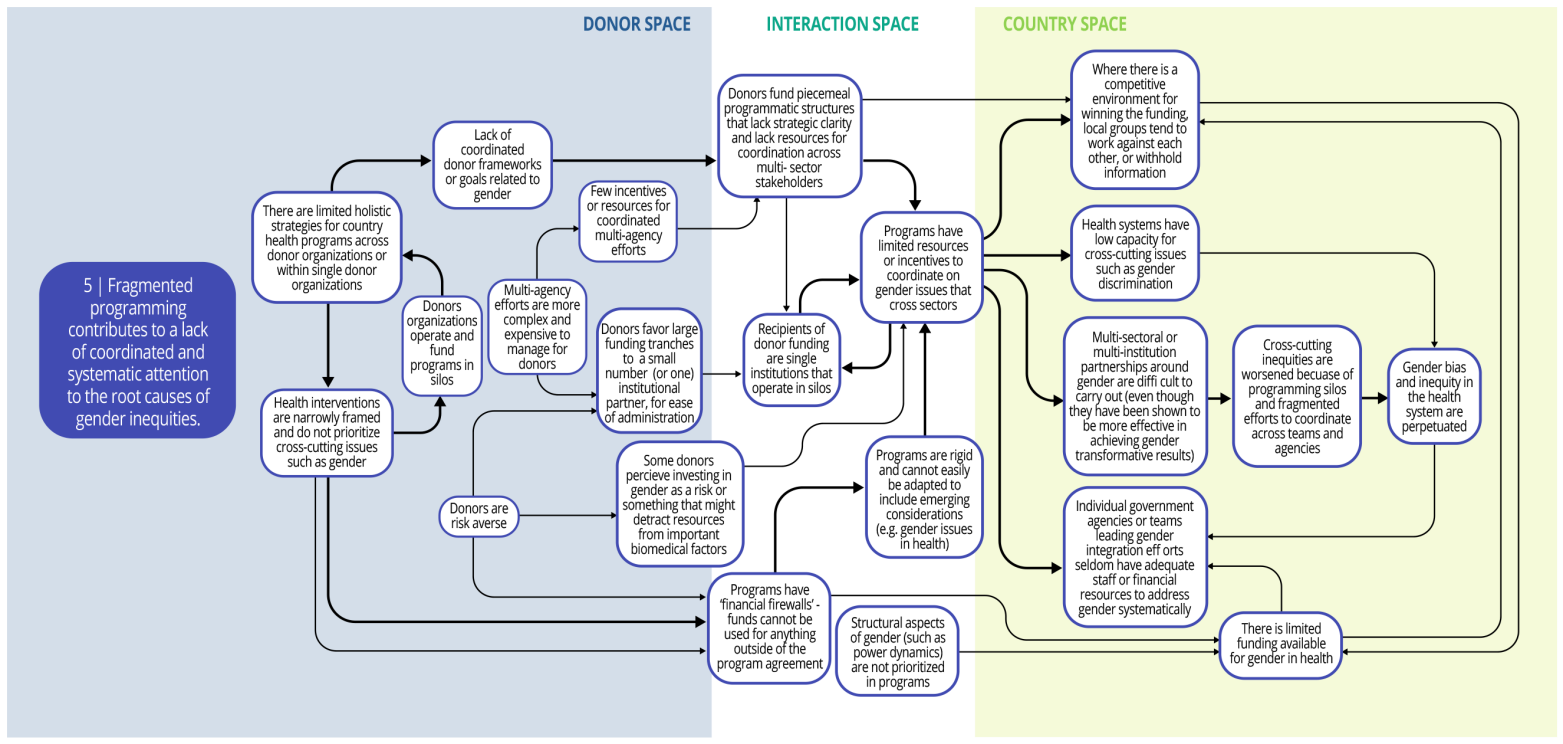
Syndrome 5: Fragmented programming contributes to a lack of coordinated and systematic attention to the root causes of gender inequities.

*Syndrome five: Fragmented programming contributes to a lack of coordinated and systematic attention to the root causes of gender inequities (see Figure 7)*

Despite significant efforts to achieve better coordination, fragmentation is an enduring feature of health financing and programming. In general, coordinated frameworks or goals related to gender inequities among and across donor organizations are lacking, which contributes to a deprioritization of gender as a crosscutting issue. Donor-funded health programs tend to have limited resources for coordination across sector stakeholders for crosscutting issues like gender inequity, perhaps because they are more difficult for donors to administer. Large funding tranches available to a few competitors can incentivize organizations or consortium groups to work against each other or withhold information, further impeding collaboration and coordination. Government agencies and teams leading gender integration efforts across health or other sectors seldom have adequate staff or financial resources for such coordination efforts, leading to unsystematic attention to gender inequities. Despite this, DAH programs rarely focus on fixing these crosscutting and coordination challenges.

**Figure 8.**
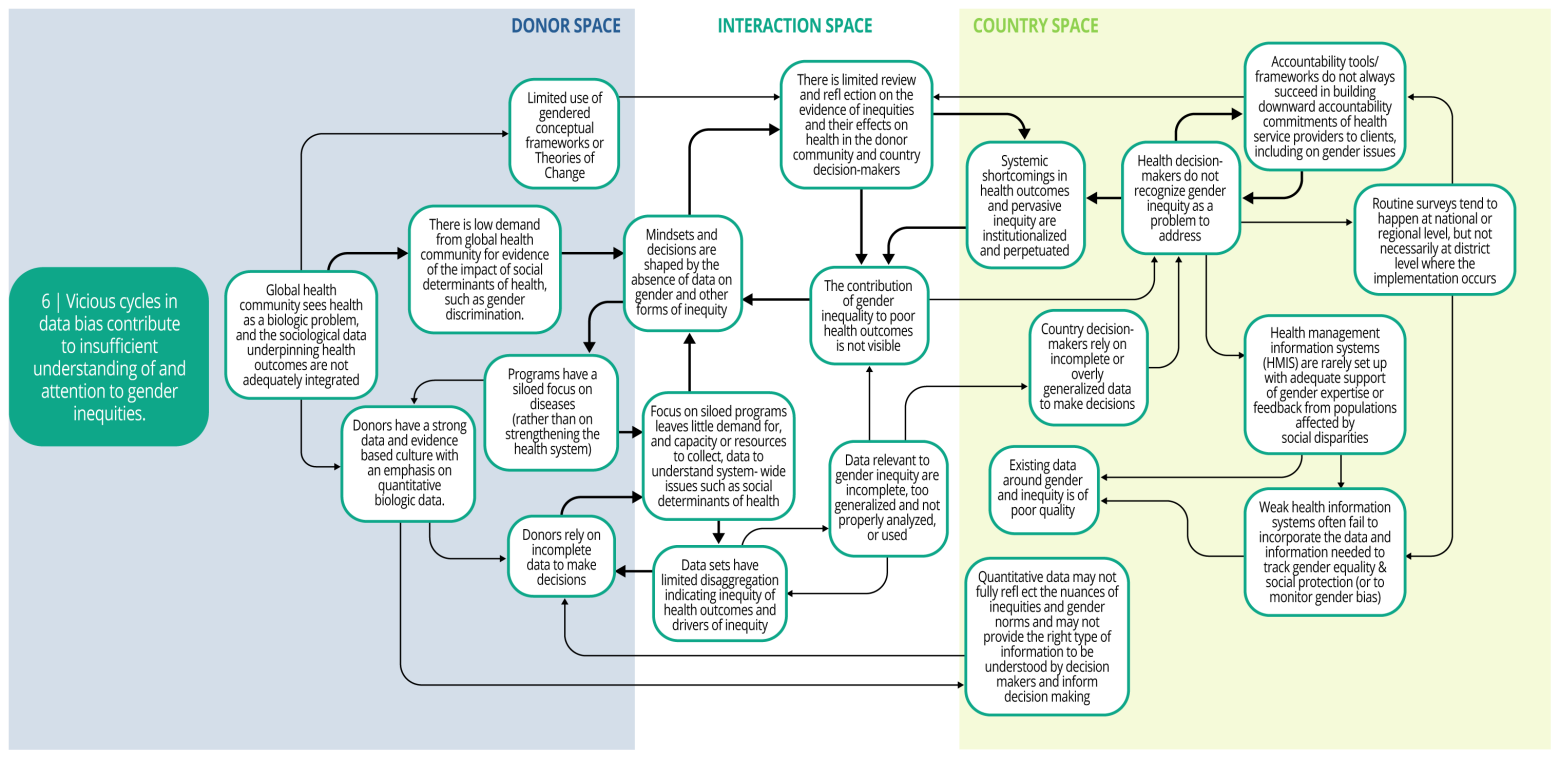
Syndrome 6: Vicious cycles in data bias contribute to insufficient understanding of and attention to gender inequities.

*Syndrome six: Vicious cycles in data bias contribute to insufficient understanding of and attention to gender inequities (see Figure 8)*

Donors, national policy makers, and health technocrats may rely on incomplete or overly generalizable data to make decisions, which ultimately perpetuates gender and health inequities. DAH programs and national health systems have common data weaknesses. Emphasis on siloed programs in global health that focus on singular health or disease areas hinder capacity and resources to understand and overcome system-wide challenges like gender inequity. In national health information systems, data are rarely collected and disaggregated in ways that provide nuance to reveal experiences of inequities and their contribution to poor health outcomes. Data relevant to explaining gender and health inequities (e.g., son preference, women’s mobility and ability to make decisions about their own bodies) may be overlooked by donors or country technocrats when making decisions about health investments. There is also a preference for, and overreliance on, quantitative over qualitative data, which can limit understanding of gender dynamics. Furthermore, health programs lack input and feedback from populations affected by gender and health inequities, or other gender experts, about whom data are important for decision-making. These factors contribute to insufficient recognition of the role of gender inequities in poor health outcomes, and thus, to the perpetuation of gender and health inequities.

## Discussion

This initiative convened donors and country actors for a systems-based diagnostic process to examine the interconnected drivers of gender inequities within the complex dynamic system of DAH, with a particular focus on the donor space. The findings highlight a set of six thematic areas (named as syndromes) impeding progress in addressing gender discrimination and bias within the DAH landscape. These six areas can be characterized as systemic in nature, as they manifest within the institutional structures and practices of DAH, as well as in the dynamic spaces of interaction between system actors. Few studies have formally examined gender in the power dynamics across a complex adaptive system such as DAH. This study provides a unique opportunity to explore these system drivers of gender inequity in DAH and inform gender transformative ^i^ opportunities for action, as called for in the critical shifts.

### Power asymmetries in development assistance for health

A leading driver across the six syndromes is the manifestation of power asymmetries. In complex adaptative systems like DAH, power manifests in many ways and in all levels of the system ^54^. There is growing recognition that power asymmetries in DAH are contributing to disparities in health outcomes ^30, 33, 55^.

Our findings depict how, despite an explicit goal to improve health and health equity in low- and middle-income countries, at a systems level, DAH appears to be structured to implicitly maintain power over, and distance from, people most affected by gender inequities—the very people it aims to serve. The findings illustrate a systematic omission of DAH mechanisms by which local individuals, groups and NGOs advancing gender equity can meaningfully participate in and lead DAH-funded initiatives, despite calls for localization of aid. The roots of this systematic omission can be seen across the six syndromes, demonstrating that interventions to gain improvements will require holistic approaches.

As illustrated in syndromes 1, 3, 4 and 5, and confirmed in the literature, DAH is structured in a way that reinforces power asymmetries, through top-down models, rigid templates, requirements, and timelines that limit genuine engagement and co-creation with local stakeholders who have the requisite gender expertise and lived experience for that setting ^56– 58^. The findings show that these models also tend to result in insufficient timelines and budgets to meaningfully address gender inequities. Furthermore, Syndromes 1 and 4 highlight how donor funding constraints and regulations contribute to reducing civil society’s role to service delivery and health promotion, rather than much-needed accountability and advocacy roles ^59^. Conceptual and operational models, decisions, and information flow in global health institutions can also reinforce power dynamics that impede progress in effectively addressing gender and health inequity ^18, 35^.

Another illustration of power asymmetries is found in Syndrome 2, which explores multifaceted bias within institutional health paradigms, practices, beliefs and decisions that perpetuate gender disparities and bias. Donor mindsets and perceptions of local capacity have been shown to play a role in how health funding is structured to exclude local civil society actors from meaningful roles in leadership, problem identification, managing resources, or evaluation of health programs ^29, 61^. Studies suggest that such patterns may stem from the high levels of representation of groups with societal, historical and educational privilege among leaders in global health organizations, who can lack insights into the realities of gender and other forms of discrimination in low- and middle-income countries ^29, 60^. The findings demonstrate that approaches to overcoming gender inequities in DAH must go beyond checklists or prescriptive approaches, and address deep-seated bias and representation in DAH institutional culture, practices, and leadership.

Syndrome 6 illustrates how limited demand for, and use of, data on gender and other intersectional experiences of bias and exclusion also contribute to reinforce power asymmetries and gender and health inequities. This lack of holistic data across DAH limits the visibility of the needs and participation of client groups affected by health disparities ^62, 63^. Health initiatives that lack data on gender and other intersectional factors are more likely to reinforce multiple levels of bias and discrimination, and negatively affect both life-course and health ^64^. In particular, the findings in Syndrome 6 highlight how donor funding can incentivize and reinforce inadequate data collection and analysis processes in health systems. This suggests a novel opportunity for donors seeking to improve their own gender transformative interventions to apply an intersectional lens in the collection, analysis and use of data in donor-funded health programs.

Overall, these findings support arguments that the drive toward progress on gender equity in DAH is a political project more than a technical one, requiring shifts in power and relationship dynamics at micro and macro levels ^16, 35, 65^.

### Disrupting the syndromes

To disrupt the syndromes and advance gender transformative programming, the future model of DAH must be fundamentally reoriented to function with, for, and led by groups affected by gender inequities. Drawing on the dynamics and barriers highlighted in the syndromes, we have highlighted five key areas for action. These are presented as preliminary areas for consideration, rather than prescriptions. Co-creation of solutions that transform the system will require further coordinated analysis, dialogue, and action in each context. Where available, corresponding promising practices, drawn from the literature, are also presented. 

***Reflect on institutional biases and move toward approaches that shift or share decision-making power***

There are growing calls for global health institutions to face their own biases, shift mindsets of privilege, and adopt practices that correct power imbalances ^66^. We urge decision-makers in DAH to question their own assumptions, through reflexive spaces, seeking answers to such questions as:

How are my biases, attitudes and beliefs influencing my opinions and actions? How does my privilege directly or indirectly disadvantage others? What can I do to address this?

Such processes are not amenable to checklists or prescriptive approaches, but require transformational learning spaces, which include both safe spaces to talk about personal biases, cultural beliefs, and practices, and endorsement of the work by leadership. Several resources exist to support such efforts ^67– 69^.

Additionally, DAH needs to move beyond the strictly biomedical paradigm that enables discriminatory health practices. To do this, donors and other global health institutions need to prioritize approaches to health that build on context-specific knowledge and values, with spaces for reflexive learning and dialogue that welcome diverse voices ^70^.

There is also a need to reform and restructure the donor-recipient relationship. Shifting power dynamics between donors and grantees requires recognizing their complementary skills, expertise, and interdependence in achieving common objectives ^71, 72^. Mechanisms for candid reflection and shared learning, trust-building, and mutual accountability are important components of this. One such approach is a mechanism for confidential and anonymous feedback ^73, 74^. Beyond engagement, some donors are embracing participatory grant-making models that aim to shift or share decision-making power about funding. These range from building in more representation of affected groups as advisors and funding decision-making bodies to ceding decision-making power about funding strategies and criteria to the communities and groups that funders aim to serve ^75– 77^.

***Create leadership and funding opportunities for groups most affected by gender and health inequities***

We urge decision-makers in DAH to create formal and systematic avenues for the leadership of grassroots civil society groups focused on gender issues in health program design, implementation and evaluation. This can improve the contextualization of issues that may be missed in standard gender assessments, and ensure that projects are relevant, responsive to the needs of participants, and sustainable ^56, 61^.

Where avenues for leadership by civil society groups do not exist, donors can support civil society engagement mechanisms as a foundational step. Such mechanisms can reveal issues of unintended harm or opportunities for program modifications that improve effectiveness ^61^. Some donors have implemented community advisory committees in their grant-making processes, which offer a formal platform for transparency about a funder’s plans at the country level and for members of affected populations and civil society organizations that represent them to provide input about the proposed interventions ^56, 74, 75^.

***Increase and restructure funding to gender and health equity advocates and stakeholder groups, including local women’s organizations***

We call upon donors to increase accessibility of funds to local groups working to achieve health and gender equality. Donor funding that enables collective efforts by country-based or regional health and gender coalitions has been shown to facilitate successful efforts to address gender inequities and achievement of outcomes ^11, 56, 72, 78^. Mechanisms for increasing accessibility include offering different funding tranche sizes to accommodate needs and capacities of local groups; structuring funding so that it can be accessed by gender advocacy networks, coalitions, and cross-sector working groups; structuring funds in a way that supports core funding; being responsive to the needs of grantees and adaptable to a changing political context; and building in adequate timelines and resources ^56, 72, 78, 79^. When unable to provide grant funding directly, donors should consider re-granting and other flexible mechanisms that allow funds to be allocated from larger institutions to smaller groups ^57, 72^.

Furthermore, donors can structure funding for planning and exit strategies in ways that build sustainability for gender-focused civil society groups to engage with government health counterparts through specific planning, catalytic, bridge, or exit grants ^57, 72, 80, 81^. Beyond greater support for local civil society actors, donors should fund crosscutting governmental institutions tasked with integrating gender ^11, 18, 82^.

***Implement coordinated approaches to reduce fragmentation of gender efforts***

We call on donors to finance and convene platforms for demand-driven multi-stakeholder co-learning, including groups most affected by gender and health inequities. Case examples of funding modalities that enable multi-institution efforts to address gender and health inequities, such as supporting networking and coordination across diverse social movement actors, demonstrate how donor funding can play a role in enabling efficacy and sustainability of program outcomes ^56, 73, 79^.

We encourage donors to learn from existing efforts to restructure funding to overcome the challenge of fragmentation in gender and health efforts. For example, the government of Ireland has established standard resources for ensuring cross-sectoral linkages across partners and government sectors on gender issues ^83^. The government of Switzerland’s approach includes basket funding for gender-related activities ^83^. Other donors have opted to create pooled funding mechanisms via multi-donor collaborations that incorporate incentives for harmonized efforts in addressing gender ^72^.

***Generate and improve access to complete, reliable, and useful information for addressing gender and health inequities***

Donors can support improved gender and health equity outcomes by applying a more robust intersectionality lens in the measures, processes, and accessibility of health data and information. Stronger collection and analysis of data on structural and systemic factors of bias, discrimination and exclusion will facilitate a deeper understanding of how interventions work and how to evaluate system-wide efficacy ^84, 85^. It has been argued that applying a more robust intersectionality lens to health data may be the key to addressing persistent health inequalities ^86^.

The World Health Organization has partnered with national governments to strengthen capacity to analyze which constituents are missing from health service data and why ^87– 90^. Such tools can help donors and health policy makers set priories by identifying the largest health inequities within a country. However, more in-depth measures and tools are required to explain why inequalities exist. Better measures are needed to examine who has what (access to resources); who does what (division of labor and everyday practices); how values are defined (social norms); who decides (rules and decision-making); and who benefits ^91^. Donors are called to support research to design health-related measures that can be used to assess structural elements of power and inequity (such as gender norms, policies, and institutional practices), beyond individual aspects of discrimination ^92^. Beyond incorporating more explanatory gender and intersectionality measures, donors are called to improve their mechanisms for gathering and using data to make decisions. Donor funding that enables civil society advocacy groups to access and translate health information for policy makers has been shown to support improved health services ^73^. Models with more inclusive methods of data collection and open data sharing are showing promise in supporting a more equitable data landscape ^87, 93^.

### Limitations

The findings presented in this paper were informed by a co-creation process to develop a shared understanding of system drivers of gender inequity in DAH. The results are therefore shaped by the perspectives and insights drawn from the lived experiences of the initiative participants and are not exhaustive or representative of all contexts. For instance, we were not able to engage a wide cross-section of representatives across geographic areas or linguistic backgrounds. While constituents of community groups most affected by gender inequities in low-income countries were represented by participating civil society organizations advocating for women’s issues, we were not able to directly include community voices. This was mainly due to language, time constraints, and the virtual format. More co-creation exercises with diverse community-based stakeholders are needed.

The co-creation process was virtual due to the COVID-19 pandemic. While online workshops can enable greater global participation by reducing geographic, cost and time barriers for participants, they also expose other power dynamics such as the digital divide between academic partners and community co-researchers. Online participation requires the skills and ability to utilize different software, stable internet connection, and access to digital devices; these prerequisites do not always exist in resource-constrained settings. In addition, the group dynamic changes in a virtual context, often inhibiting the level of relationship-building and trust-forming that can happen face-to-face. The IAWG Secretariat tried to mitigate technical barriers to participation by reimbursing for internet fees, however, the virtual environment likely hindered the level of rapport built between co-creation participants and facilitators.

The literature review was limited in depth and scope. A broader scoping of the literature, including articles in other languages and formats would capture a more diverse set of voices and insights.

The scenarios depicted in the syndrome maps should not be interpreted as an absolute or holistic view of how gender inequity manifests, nor do they reflect the nuances of individual donor modalities or country or community contexts. Rather, the specific drivers and dynamics portrayed in the syndromes are examples of underlying factors of gender inequities in a highly dynamic and complex system.

### Areas for additional exploration

This initiative explored systems dynamics affecting gender inequities in health, with a particular focus on the donor space. The six syndromes represent an overview of the drivers; each syndrome would benefit from further analysis. In the dynamic complexity of DAH and global health, a fuller conceptualization and analysis of gender and power, drawing on insights from community members, civil society organizations, implementing partners, and staff and custodians of national health systems is needed. More research on dynamics in the coordination and collaboration spaces between civil society and health system actors that drive gender inequity is needed. An analysis of institutional culture and leadership would be useful to find opportunities for more equitable and inclusive structures for grant-making and health service delivery. Modalities to understand and address gender inequities manifested in national health systems will be vital for improving gender and health equity. Further studies of efforts to improve accountability to achieve more equitable and inclusive DAH strategies are also needed. More research is needed to explore how gender and other social determinants of health are conceptualized, measured and analyzed in health data, and how social justice approaches to intersectionality can be better applied.

## Conclusions

Our findings present a novel perspective on systemic challenges in DAH that perpetuate or contribute to gender inequities, with a particular focus on the role of donors. The findings emphasize that many of the barriers to gender equity in DAH are embedded in unequal power dynamics that distance and disempower those most affected by gender inequity in the very programs intended to help them. Overcoming these dynamics will require more than technical solutions. To advance progress in gender equity in global health, and specifically DAH, leaders (including donors, ministry representatives, health technocrats, and those implementing health programs) must apply tools and processes that center groups affected by gender inequity in leadership and decision-making at micro and macro levels. This should include building practices and structures that enable co-creation and mutual accountability in the design, implementation, and evaluation of health programs.

An important feature of this effort was convening a diverse set of stakeholders to examine a common problem. The shared dialogue provided nuanced insights on why progress addressing gender inequity has been slower than hoped, despite attempts to do so in health programs. Such platforms for cross-stakeholder dialogue are, in themselves, promising for future gender equity endeavors.

## Data Availability

Zenodo: Exploring system drivers of gender inequity in development assistance for health and opportunities for action. https://doi.org/10.5281/zenodo.6612438 ^94^. This project contains the following underlying data: Draft Syndrome maps_compiled.pdf. (System maps of original syndromes using Miro virtual whiteboard tool to synthesize findings from first phase of data collection, i.e., co-creation workshops. Combined into one PDF page.). Draft Syndrome Maps_individual.pdf. (System maps of original syndromes using Miro virtual whiteboard tool to synthesize findings from first phase of data collection. Each syndrome listed in a separate map.). Raw Data_ April 2021 Co-Creation Workshop.pdf. (System mapping inputs gathered during virtual co-creation workshop April 21-22, 2022 using Miro virtual whiteboard tool). Raw Data Gender Syndrome Dialogue Sessions_updated.pdf. (Updated system maps of syndromes using Miro virtual whiteboard tool during two group discussions in the second phase of data collection/iteration. The calls were held with co-authors on November 29 and December 8, 2021). Raw data_KII_02.pdf. (Key Informant Interview Transcripts). Raw data_KII_03.pdf (Key Informant Interview Transcripts). Data are available under the terms of the Creative Commons Attribution 4.0 International.

## Footnotes

^a^DAH, as defined by the Institute for Health Metrics and Evaluation, refers to “Financial and in-kind resources that are transferred through major international development to low- and middle-income countries with the primary purpose of maintaining or improving health.” ^34^

^b^We use the term DAH landscape to refer to the actors, norms, and structures that interact in the context of external financial contributions for activities aimed at improving health in low- and middle-income countries.

^c^Throughout this paper, we use the term donor to denote institutions that provide external funding for health. We recognize the varying terminology and labels—including development partners, funding partners, external funding, lending institutions, and investors—and approaches across those providing funding for health, and acknowledge the nuances and connotations of these terminologies. For our purposes, we continue to use the term donor for clarity and consistency, as this is how we framed the conversation with co-creation participants throughout the process.

^d^This approach combines multiple theories and concepts ranging from systems theory ^43, 44^, innovation and diffusion theory ^45^, design thinking ^46^, and resilience theory ^47^.

^e^The IAWG was formed in 2020 by representatives from the United States Agency for International Development, The World Bank, and the Bill & Melinda Gates Foundation to examine and advance the critical shifts to redistribute power and achieve more effective health outcomes. JSI Research and Training Institute, Inc., in partnership with Global ChangeLabs, served as the IAWG secretariat.

^f^For more information on Figure 1 (name) see this brief: https://publications.jsi.com/JSIInternet/Inc/Common/_download_pub.cfm?id=25296&lid=3

^g^Social constructivist approaches are grounded in the idea that learning is a collaborative process. Research processes using social constructivist approaches are based on the idea that understanding something better comes from interaction between people, and the historical, cultural, and social context within a specific period of time. This is in contrast to positivist approaches, which assume there is only one valid reality that can be “discovered” and “made known'' through objective research ^50, 51^.

^h^To support co-creation and allow simpler annotation of the system maps to facilitate legibility, the System Acupuncture® process intentionally frames the drivers to be valence-aligned to reflect barriers to or inhibitors of achieving desired outcomes. This reduces the need to annotate reinforcing or counteracting relationships between drivers, as most causal relationships will be correlated positively.

^i^‘Gender transformative’ refers to policies and programs that seek to transform gender relations to promote equality and achieve program objectives by: 1) fostering critical examination of inequalities and gender norms, roles, and dynamics; 2) recognizing and strengthening norms that support equality and an enabling environment; 3) promoting the relative position of women, girls, and marginalized groups; and 4) transforming the underlying social structures, policies, and broadly held social norms that perpetuate gender inequalities ^53^.
