# Supplementary material for: Exploring system drivers of gender inequity in development assistance for health and opportunities for action
Source: Gates Open Res. 2023 Jul 17;6:114. Originally published 2022 Aug 24. [Version 2] doi: 10.12688/gatesopenres.13639.2 (PMC10427755; doi:10.12688/gatesopenres.13639.2)
Supplement: Peer review history

# Peer review: Reviewer response for version 2

**DOI:** 10.21956/gatesopenres.16122.r34264

**Published:** 2023-08-15

**Authors:** Roopa Dhatt

**Affiliations:** 1 Women in Global Health, Hayward, CA, USA; 2 Georgetown University, Washington, District of Columbia, USA

The authors have sufficiently addressed the concerns expressed. They have made improvement to the article.

Is the work clearly and accurately presented and does it cite the current literature?

Yes

If applicable, is the statistical analysis and its interpretation appropriate?

Not applicable

Are all the source data underlying the results available to ensure full reproducibility?

Partly

Is the study design appropriate and is the work technically sound?

Yes

Are the conclusions drawn adequately supported by the results?

Yes

Are sufficient details of methods and analysis provided to allow replication by others?

Partly

Reviewer Expertise:

Health workforce, women's leadership in health, gender equality in global health, gender transformative leadership, health in all policies, political economy

I confirm that I have read this submission and believe that I have an appropriate level of expertise to confirm that it is of an acceptable scientific standard.

# Peer review: Reviewer response for version 1

**DOI:** 10.21956/gatesopenres.14918.r32832

**Published:** 2023-02-10

**Authors:** Roopa Dhatt

**Affiliations:** 1 Women in Global Health, Hayward, CA, USA; 2 Georgetown University, Washington, District of Columbia, USA

1. Methodology: In the desk review, in addition to seeking articles on 'gender', was there an attempt to seek out articles about women? The majority, if not all, women are underrepresented in leadership roles, have less power in health systems, so seeking out articles than aim to support women is needed ensure complete data collection. 
2. Define co-creation. 
3. The word choice syndrome seems a bit odd. Although explained in the paper, including the use of the metaphor. It appears far reaching and confusing to use syndrome in this setting. 
4. Each of the Syndromes - should be changed to a phenomena and have specific headings. 
5. Utilize gender transformative approaches in this paper, as getting to the root causes and working to address these root causes through addressing power imbalances is what gender transformative leadership is about. Reference: https://www.devex.com/news/opinion-a-new-vision-for-global-health-leadership-93772 

Is the work clearly and accurately presented and does it cite the current literature?

Yes

If applicable, is the statistical analysis and its interpretation appropriate?

Not applicable

Are all the source data underlying the results available to ensure full reproducibility?

Partly

Is the study design appropriate and is the work technically sound?

Yes

Are the conclusions drawn adequately supported by the results?

Yes

Are sufficient details of methods and analysis provided to allow replication by others?

Partly

Reviewer Expertise:

health workforce, women's leadership in health, gender equality in global health, gender transformative leadership, health in all policies, political economy

I confirm that I have read this submission and believe that I have an appropriate level of expertise to confirm that it is of an acceptable scientific standard, however I have significant reservations, as outlined above.

# Response

**Published:** 2023-06-23

**Authors:** Sita Strother

**Affiliations:** JSI, USA

Thank you for these comments and suggestions. Please see below details on how we have addressed your comments (numbered according to your original comments):

1. The desk review included numerous articles citing gaps in women's leadership within the health sector workforce and in other DAH stakeholder institutions. See references 8-10,12, 16, 17, 29, 60. 
2. Thank you, we have added a definition for the term 'co-creation' to the Methods section.
3. The term "syndrome" is used in the Systems Acupuncture method, and has been used in the two corresponding manuscripts. We opted to keep it here for consistency.
4. The syndrome headings are framed to illustrate the phenomenon and its effect. The descriptive paragraph associated with each syndrome provides a detailed description of the map graphics. 
5. We have applied a gender transformative analytic lens in this paper. Our findings cite root causes of gender and power imbalances, and we call for actions that address systemic inequities in DAH, with implications for power shifts and political solutions at micro and macro levels.

# Peer review: Reviewer response for version 1

**DOI:** 10.21956/gatesopenres.14918.r32841

**Published:** 2023-02-02

**Authors:** Rosie Steege

**Affiliations:** 1 Liverpool School of Tropical Medicine, Liverpool, UK; 2 London School of Hygiene & Tropical Medicine, London, UK

I would like to thank the authors for their innovative and comprehensive analysis of the barriers to gender equity in DAH. It is a unique angle and a valuable contribution to the literature on structural gender inequities. The paper is well written, engaging and I enjoyed the metaphor of syndromes. With each syndrome I found myself nodding along in recognition of these barriers, which I have seen reflected time and time again. The diagrams and visuals are nice (though each bubble is hard to read so the authors may wish to consider laying these out in full size as a supplementary file). I like the inclusion of solutions and recommendations and practical questions for donors to consider.

 I am in support of this paper and it’s contribution to the literature, there are however, three key points I would like to see addressed to strengthen the paper:

- The paper discusses the need for co-creation and inclusion of community voices ‘nothing about us without us’. This wasn’t done as part of this research which makes it seem disingenuous, although it is briefly mentioned in limitations. I do appreciate the limits of conducting this type of research and the value of this paper remains. Perhaps you could more explicitly address why this wasn’t done and be more upfront about it. Including the voices of those communities you wish to centre is obviously key, so perhaps the authors could consider a validation exercise with these groups, or a follow on co-creation workshop, considering it’s centrality to your argument?
- Secondly, the issue of donor power in financing as highlighted in syndrome 3 is critical and links to accountability. How individual donors have the power/resources to shape the DAH landscape is also remarkable (and there are links here to the literature on equitable partnerships and funding streams). I would like to see this acknowledged within the paper given the authors’ links to the Gates Foundation. Maybe there are unique initiatives that the Gates Foundation are employing here that the authors could draw upon and share as examples of ‘best practice’ or if not, why not? Also considering the topic of the paper and given these links it would be critical to include some reflection on the positionality of the authors within the methods and even, at the end as per Morton *et al.*’s consensus statement – see Morton *et al. *(2022 ^1^).
- Finally, the methods are innovative and novel and I’m sure will be of interest to many. Having just been involved in running a similar co-creation workshop approach myself I am interested to learn more about the methodology. Currently, however, I don’t feel it is well enough described for those who wish to employ these methods themselves. When systems acupuncture is first mentioned there is a small footnote to help explain that you may wish to bring into the body of the text and then again in the methods set out more clearly what the approach is. If I wanted to employ the method, how might I go about it? It reads like a desk review and then co-creation workshop but with maps making it distinct - were the maps created live during the workshop and then synthesised after by core study team, or were sub-groups involved in this process? I also interpret it that the challenges were identified in the workshop, and the recommendations by authors, but please clarify.

**Specific other points by section:**

Introduction:

- I would say that the terms female/male relates to sex, but that women/girls/men/boys relates to gender.

Methods:

- Ethics for the KIIs isn’t made clear in the methods – please include information on the ethics, and how these experts were recruited.
- The validation was done via key informant interviews – was there also validation exercise done with the other workshop participants?
- How did the workshop participants contribute to the analysis or synthesis?

Results:

- Were there any findings on the proliferation of men in decision making roles limiting progress? Evidence has shown that gender is often conflated with women’s issues – so for example in the SASA! Intervention to reduce IPV they purposefully avoided the word gender so that men remained engaged, I wonder if these things are being replicated at higher levels of the health system and donor space?

Discussion:

- You call for local CSOs and women’s organisations to be included, were these included in your co-creation workshops?

- You discuss the important need for data for decision making. I would argue we also need indicators that support an intersectional analysis. This data is limiting tailored and equitable approaches to gender transformation as axes of inequity come together to shape marginalisation in different ways.

Limitations:

- The literature review was non-systematic, do you feel this was a limitation? What languages were included in your review, seeing as knowledge generation is often skewed to global north?
- It would be good to include reflection on how the virtual process of co-creation workshops have excluded or included perspectives in knowledge generation? See Egid *et al.* (2021 ^2^) for interesting reflections.

Is the work clearly and accurately presented and does it cite the current literature?

Yes

If applicable, is the statistical analysis and its interpretation appropriate?

Not applicable

Are all the source data underlying the results available to ensure full reproducibility?

Yes

Is the study design appropriate and is the work technically sound?

Yes

Are the conclusions drawn adequately supported by the results?

Yes

Are sufficient details of methods and analysis provided to allow replication by others?

Partly

Reviewer Expertise:

gender equity; intersectionality; health systems; co-production; equitable partnerships

I confirm that I have read this submission and believe that I have an appropriate level of expertise to confirm that it is of an acceptable scientific standard, however I have significant reservations, as outlined above.

## References

[1] : Consensus statement on measures to promote equitable authorship in the publication of research from international partnerships. *Anaesthesia* .2022;77(3) : 10.1111/anae.15597 264-276 10.1111/anae.15597 34647323PMC9293237

[2] : Can everyone hear me? Reflections on the use of global online workshops for promoting inclusive knowledge generation. *Qualitative Research* .2021; 10.1177/14687941211019585 10.1177/14687941211019585 PMC1036166637485303

# Response

**Published:** 2023-06-23

**Authors:** Sita Strother

**Affiliations:** JSI, USA

Thank you for this thorough review. Please see below details on how we have addressed your comments (grouped according to section of the paper):

- **Addressing why community voices were not included:** The time, scope, and budget constraints of this initiative affected the breadth of voices included. Because of the shift to a virtual format due to the COVID-19 pandemic, we were not able to directly include community voices in the co-creation workshop. While we were able to include civil society members with internet connectivity who represented women's groups, we recognize this as a key limitation to this work, and have clarified this point in the limitations section.
- **Reflections on donor power and positionality of the authors**: We have expanded on  the role of donor power in the discussion section, which also includes examples of how some donors are addressing this power dynamic. We have also added a positionality statement to the Methods section. A separate paper stemming from this initiative also explores donor power within the broader DAH landscape (See reference 43). 
- **Clarifying the methods**: We have updated the methods section to clarify the processes we followed. In addition, the workshop outputs are available to those interested in more details (linked within the paper). 
- **Introduction**: We have updated our definition in the intro for greater clarity. 
- **Methods**: 1) We have clarified the KII ethics in the ethical statement. 2) Regarding the validation process, this has been clarified in the 'process steps' sub-section of the Methods. We have also added more detail on stakeholder engagement throughout this process  (see Fig 2 and sub-section on 'co-creation and participant engagement'). 3) Workshop participant role in analysis/synthesis has been clarified in the 'process steps' subsection of the methods. See also Figure 2.
- **Results**: Co-creation workshop participants raised the issue of limited representation of women and marginalized groups in decision-making roles. This, and the related issue of privilege bias is represented and discussed most prominently in Syndrome 2. 
- **Discussion**: 1) Role of local CSOs and women’s organizations in the workshops — This has been clarified in the methods and limitations section. 2) Intersectional analysis — We have incorporated a clearer call-out to the role of intersectional factors and the need for an intersectional approach to data collection, analysis and use in health programs in the discussion section. Refer to the updated sub-sections on 'power asymmetries...' and 'disrupting the syndromes'.
- **Limitations**: 1) The methods section acknowledges that the literature review was non-systematic; we have added a statement in the limitations around only reviewing English-language articles. 2) Thank you for pointing us to Egid et al, which is highly relevant to our methods and limitations. We have added a paragraph discussing the power dynamics exposed by the virtual workshop environment to the limitations section.

# Peer review: Reviewer response for version 1

**DOI:** 10.21956/gatesopenres.14918.r32843

**Published:** 2023-01-30

**Authors:** Katherine Hay

**Affiliations:** 1 Center on Gender Equity and Health, Division of Global Public Health, University of California, San Diego School of Medicine, La Jolla, CA, USA

This is a thoughtful and creative paper from a diverse and interestingly placed set of authors. It explores how relationships and dynamics in donor assistance perpetuate and reinforce power and gender asymmetries and inequities. It is detailed, well written, includes multiple perspectives, and adds value and richness to the field and discussion. It uses novel methods and is a call to action.

 The majority of my questions are around clarifying methods and analysis. As some of these methods seem fairly novel I hope by adding in a little more detail it will also help others who may be interested in exploring some of these approaches in the future. 

 I am very supportive of this paper and hope the authors can easily address my comments.

1. As I understand it, their primary data (the development of the syndromes) is based on key insights from 6 key respondents. It would be helpful to clarify that none of these key informants are also the authors.   
2. It would also be helpful to include in the discussion some analysis on how the perspectives of these particular 6 informants was triangulated and used to form the maps. For example was each word bubble the statement of one person or a set of triangulated statements?
3. Please clarify the role of the 6 informants in the analysis, creation of the syndromes, and role (if any) in the discussion, and creation of recommendations.
4. I suggest the discussion section be reworked. For example the statement, "the maps do not reflect how challenges manifest in all cases". Which cases? From cases described in the literature review? This is not clear. I think what this section is attempting to do (and would be helpful) is to point out how the insights from the 6 key informants map against findings from the literature review, and speak to the similarities and differences. I think that could be done more clearly. What was similar, what was different? 
5. It is not always clear whether the discussion is referencing the primary data (laid out in the Syndromes) or the literature review. I suggest using the discussion section to analyze the new insight they have derived from this research and from using "Systems Acupuncture”; what has it added to our knowledge, what do they see as the limitations or strengths?  
6. The discussion section then moves into a set of recommendations. Please clarify how these recommendations were generated. In the paper you indicated that the methodology used, Systems Acupuncture, is unique because it “identifies opportunities for system level transformation.” It was not clear to me if the 5 recommendations that were generated were done so using that methodology (and if so – please describe how, and whether the same 6 key informants were involved), or whether here we pivot, and the recommendations are generated from the authors, who have weighed the analysis and are now jumping off based on the data and their expertise and knowledge. If the latter, I suggest separating out your discussion of findings from your recommendations. That way the reader can see where you move from your analysis of the data, to your own interpretation and reflections on solutions. 
7. On the recommendations, I found myself going back to the original Figure 1 to try to map back what was new or different in the recommendations from the original call to from the IAWG. I suggest you consider this as well. This feels left unconnected.
8. Smaller point. I note that Fig. 1 does not have an original citation in the text. The text suggests it was an earlier (and broader?) set of contributors who developed this so please check on the correct referencing and authors for this visual.
9. **Other points to consider:** The Syndrome maps are not readable in the print outs or the power point versions. To read  one has to expand the pdf on screen to 400% and read them essentially 1 bubble at a time. That is a barrier for most readers. As this is the bulk of your new data I would encourage you to try to think about how to share this data in a way that is more easily readable and accessible. 
10. I really liked the metaphor of the Syndrome. I found it told a story about the behaviors you were trying to explain while not overstating any particular behavior within the set. However, for the same reason I found the use of driver confusing, given that I considered your sample of 6 to be more about generating patterns then drawing casual connections. While I recognize this is disciplinary, drivers for many of your readers have a causal and relational meaning that the behaviors described in the bubbles did not have. You used many descriptors for you model. I thought "dynamics, behaviors, and characteristics," worked better to describe the content of the bubbles than drivers. Just something to consider.

Is the work clearly and accurately presented and does it cite the current literature?

Yes

If applicable, is the statistical analysis and its interpretation appropriate?

Not applicable

Are all the source data underlying the results available to ensure full reproducibility?

Yes

Is the study design appropriate and is the work technically sound?

Yes

Are the conclusions drawn adequately supported by the results?

Yes

Are sufficient details of methods and analysis provided to allow replication by others?

Partly

Reviewer Expertise:

Gender equity and health

I confirm that I have read this submission and believe that I have an appropriate level of expertise to confirm that it is of an acceptable scientific standard, however I have significant reservations, as outlined above.

# Response

**Published:** 2023-06-23

**Authors:** Sita Strother

**Affiliations:** JSI, USA

Thank you for your thorough and thoughtful review. Please see below details on how we have addressed your comments (numbered according to your original comments):

- Comments 1-3: The primary data for this paper included input from all 41 workshop participants, with substantial input from the gender sub-group. This has been clarified in the Methods section. The Methods section clarifies the role of workshop participants and other stakeholders who co-authored the paper, including analysis and recommendations. We have added Fig 2 and a subsection on 'co-creation and participant engagement' to ensure this is clear in the paper. 
- Comments 4 & 5: We have updated the discussion section to expand on how this study contributes to the field, new insights, and areas for further exploration. 
- Comment 6: The process used to generate the co-authors' recommendations is clarified in the Methods section (see 'iteration and interpretation of the gender syndromes'). The recommendations and promising practices are now grouped under a subheading ('Disrupting the Syndromes') in the Discussion for greater clarity.
- Comment 7: This paper expands on the work of two corresponding manuscripts exploring the Critical Shifts, which provide additional insights [see references #42 and 43]. The Discussion section of this paper provides opportunities for action related to gender inequities, as called for more generally in the critical shifts.
- Comment 8: We have added a note with a reference source for figure 1.
- Comment 9: Thank you for flagging. We have revised the graphics such that they should be readable to the viewer now when they click to expand.
- Comment 10: We have opted to keep the term driver for consistency, given that this is the term used in System Acupuncture and our corresponding published manuscript also uses this term. We have included a definition of drivers in the methods section and also added clarification on this in the results.
